# RAD-seq as an effective strategy for heterogenous variety identification in plants—a case study in Italian Ryegrass (*Lolium multiflorum*)

**DOI:** 10.1186/s12870-022-03617-6

**Published:** 2022-05-05

**Authors:** Qingqing Yu, Yao Ling, Yanli Xiong, Wenda Zhao, Yi Xiong, Zhixiao Dong, Jian Yang, Junming Zhao, Xinquan Zhang, Xiao Ma

**Affiliations:** College of Grassland Science and Technology, Sichuan Agricultural University, Chengdu, 611130 China

**Keywords:** *Lolium multiflorum*, Variety identification, RAD-seq, Phylogenetic analysis, SNP markers

## Abstract

The primary approach for variety distinction in Italian ryegrass is currently the DUS (distinctness, uniformity and stability) test based on phenotypic traits. Considering the diverse genetic background within the population and the complexity of the environment, however, it is challenging to accurately distinguish varieties based on DUS criteria alone. In this study, we proposed the application of high-throughput RAD-seq to distinguish 11 Italian ryegrass varieties with three bulks of 50 individuals per variety. Our findings revealed significant differences among the 11 tested varieties. The PCA, DAPC and STRUCTURE analysis indicated a heterogeneous genetic background for all of them, and the AMOVA analysis also showed large genetic variance among these varieties (Φ_ST_ = 0.373), which were clearly distinguished based on phylogenetic analysis. Further nucleotide diversity (Pi) analysis showed that the variety ‘Changjiang No.2’ had the best intra-variety consistency among 11 tested varieties. Our findings suggest that the RAD-seq could be an effectively alternative method for the variety distinction of Italian ryegrass, as well as a potential tool for open-pollinated varieties (OPVs) of other allogamous species.

**Supplementary Information:**

The online version contains supplementary material available at 10.1186/s12870-022-03617-6.

## Introduction

*Lolium multiflorum* Lam., also known as Italian ryegrass, is one of the most important cool-season annual bunch grasses with numerous advantages, such as leafiness, multiple tillers, high herbage yield, palatability, digestibility, rapid seed establishment and weed suppression [1, 2]. Italian ryegrass is an excellent short duration grass chiefly used for pasture and silage in dairy and beef cattle production. About 635 cultivated varieties of Italian ryegrass have been registered worldwide [3]. The International Union for the Protection of New Varieties of Plants (UPOV) has developed DUS (distinctness, uniformity and stability) testing guidelines for cultivated ryegrass species (https://www.upov.int/edocs/tgdocs/en/tg004.pdf), in which 22 morphological traits (except for ploidy level) under field conditions are required to evaluate in at least 60 spaced plant and 8 m of row plot trials. With the continually rising number of new varieties released onto the market, discrimination on the basis of morpho-physiological characteristics becomes increasingly difficult and limited, due to a requirement to include larger numbers of similar varieties in the trial along with high genetic heterogeneity within cultivated varieties, thus leading to high cost, complexity and poor capacity [4, 5]. Moreover, the complexity of the environment such as temperature, rainfall, illumination and other factors affect the accuracy of DUS tests for morpho-physiological traits, as well as the assessment of a candidate variety [6]. These factors result in many challenges when achieving international intellectual property protection that should be based on highly efficient and accurate identification of large numbers of plant varieties [7]. In this context, molecular-based/assisted DUS testing has become the focus of breeders [4, 6, 8]. The breeding system of the vast majority of seed-propagated forage grasses, such as Italian ryegrass, is cross-pollination with strong gametophytic self-incompatibility [9]. In general, a bred variety of allogamous grasses is a synthetic or open-pollinated random-mating population developed from mass selection or phenotypic recurrent selection, with large variation within varieties and little genetic difference among them [10]. Compared with the single-genotype varieties of common crops, more molecular markers and population-level analysis are necessary for these open-pollinated varieties (OPVs) of outcrossing grasses.

At present, research on the implementation of molecular marker technologies assisting DUS testing in a broad range of agricultural plant species has proven widely successful, especially for cultivars with a unique pure genetic background (a single genotype). This is due to the fact that a DNA marker system could provide a much higher number of polymorphic markers compared to the number of DUS morphological traits with their strong environmental dependence, and also has the potential to include many more samples in genotypic assays [4, 5]. However, the method of identifying OPVs generally uses multiple highly heterogeneous and heterozygous individuals for each variety, which is costly and time-consuming when the number of tested varieties is large [1, 8, 11]. Therefore, for the identification of these kinds of varieties, it is better to use bulked DNA samples, which contain different individual plants from the same variety. However, for a single bulked sample, the limited number of individuals is insufficient to represent the genetic integrity of a heterogeneous population [4]. For a large number of OPVs, it is sensible to use multiple mixed samples instead of single samples [12]. In fact, researchers have reached a consensus on using mixed samples for the variety identification of cross-pollinated species [4]. In the past decades, traditional molecular markers (e.g., AFLP, SSR, RAPD, and so on) have been employed for variety identification based on mixed samples, which is beneficial for the representation of the genetic integrity of populations because of the large number of selected individuals per variety [13]. However, these traditional fingerprinting techniques often target multiple primer‐binding sites which leads to competitive or biased PCR amplification due to affinity differences between primer and targeted DNA regions. During PCR amplification, if there is only a single mixed sample with a large number of individuals, the primers cannot effectively bind to all of DNA templates, thus some fragments cannot be amplified or observed by gel electrophoresis [1, 14, 15]. Additionally, the genetic diversity within a population variety is not fully represented when only a bulked sample from a limited number of individuals is assessed by low throughput markers. Above-mentioned issues could result in heterogeneous OPVs not being properly distinguished by traditional molecular markers.

Compared to traditional molecular markers, single nucleotide polymorphism (SNP) is more common within plant genomes, making it a better tool for variety identification in modern plant breeding programs. In particular, owing to the rapid development of next-generation sequencing (NGS) techniques, especially simplified reduced-representation approaches including the two most common methods, i.e., genotyping-by-sequencing (GBS) and restriction site associated DNA sequencing (RAD-seq), the acquisition of SNP markers has recently become cheaper and more efficient. Furthermore, implementation of GBS-SNPs has been used to distinguish varieties of outbreeding species including *Lolium perenne* and *Medicago sativa* by bulked samples, based on genome-wide allele frequency within varietal populations [4, 16]. For taxa without prior genomic information, such as Italian ryegrass, RAD-seq is the better option than GBS. Andrews KR et al. (2016) also showed that unlike many other methods for generating genome-wide data, RAD-seq does not require any prior genomic information for the taxa being studied [17]; Additionally, due to the different technologies used by GBS and RAD, the consistency of size selection across libraries is critical for producing data on a comparable set of loci across samples. Inconsistency can lead to different sets of loci appearing in different libraries, resulting in wasted sequencing effort and high levels of missing genotypes [17]. Thus, RAD-seq has been a better choice for some species such as Italian ryegrass that lacks genome information. This study uses large-size mixed samples with multiple datasets to identify eleven Italian ryegrass varieties, thereby providing a reference for auxiliary identification of OPVs, especially for species whose genome information is lacking or limited.

## Materials and methods

### Materials and DNA extraction

Eleven Italian ryegrass varieties were selected, with their detailed information summarized in Table 1. 200 seeds per variety were germinated in a growth chamber and about 180 seedlings were sampled separately at three-true-leaves stage. Each variety contained three bulked samples, which were composed of independent 50 plants, i.e., totally 150 plants per variety. For each bulked sample, 50 single young leaves of similar size from each of randomly chosen 50 plants were pooled prior to DNA extraction. Total DNA extraction was carried out with a DNA Extraction Kit (Tiangen, Beijing, China) for each bulk. The quality of DNA was checked by Qubit and Nanodrop assay kits (Thermo Fisher Scientific, Waltham, MA).

**Table 1:** The Italian ryegrass varieties registered by the National Forage Varieties Approval Committee (China)

No	Variety	Pedigree	Breeding organization
YC	Yancheng	The germplasm introduced to China by the United States in 1946 were bred by mixed selection on coastal tidal flats	Agricultural Science Research Institute of Jiangsu Coastal Area
JT	Jivet	Plant Breeders Rights (breeding origin: unknown)	DLF Trifolium
ABD	Aubade	A Plant Variety Protection variety bred in the Netherlands	Imperial Valley Milling Co., USA
TG	Tetragold	Plant Breeders Rights (breeding origin: unknown)	Barenbrug, USA
BD	Abundant	Plant Breeders Rights (breeding origin: unknown)	DLF International Seeds
AGS	Angus No.1	Plant Breeders Rights (breeding origin: unknown)	DLF International Seeds
GX	Ganxuan No.1	Breeding variety from Dutch variety Burke by doubling and radiation mutagenesis	Jiangxi Animal Husbandry Technology Extension Station
DBR	Double Barrel	Plant Breeders Rights (breeding origin: unknown)	DLF International Seeds
SNF	Shangnong Tetraploid	Mixed selection of Oregon grown varieties imported from the United States after salt stress and radiation treatments	Shanghai Jiao Tong University School of Agriculture and Biology
LTT	Blue Heaven (‘Lan Tian Tang’ in Chinese Pinyin)	Plant Breeders Rights (breeding origin: unknown)	Jacklin seed by Simplot
CJT	Changjiang No.2	The offspring of the variety Ganxuan No.1 and Aubade mixed planting and open pollination were bred through multiple mixed selections	Sichuan Agriculture University

### RAD sequencing and acquisition of datasets

RAD-seq libraries were constructed using a protocol adapted from Baird et al. [18]. Briefly, after passing quality inspection, the non-contaminated and high-quality genomic DNA was digested with EcoRI (NEB, Ipswich, MA, USA) and then heat-inactivated at 65 °C. The DNA fragments were subjected to end repair and individually barcoded P1 (Primer sequence, Illumina adapter sequences, and short sequence tags) adapters were ligated onto the cut site in each sample using the T4 ligase (NEB, Ipswich, MA, USA), which were then pooled in groups and randomly sheared to DNA fragments using a Branson Sonicator (model SX 30, Branson Ultrasonics, Danbury, CT, USA). Sheared DNA was purified, eluted, and separated using gel electrophoresis, and a DNA fraction corresponding to 300–700 bp was excised and purified. After end repair, purification, and elution, the dATP overhangs were added to the DNA fraction, and then the P2 adapters were linked. The RAD-tags were acquired during the last step by PCR detection, and were sequenced using an Illumina HiSeq2000 platform by Novogene with paired-end 150 bp (PE 150) sequencing strategy. The raw data of the Illumina sequence reads were subjected to a filtration process using FASTP v0.18.0 [19], which was performed as follows: 1) the reads with unknown nucleotides (N) ≥ 10% were removed; 2) low-quality reads (Phred quality score ≤ 20, percentage of low-quality bases ≥ 50%) were trimmed; 3) reads containing an adapter we removed. Next, the clean reads were used for assembly. Before the clean data mapped, the draft genome data published in diploid *L. multiflorum* has been noticed [20]. But this draft genome is not used as the reference genome in present study because of a poor genome assembly since N50 scaffold length of is only 5 Kb and the assembly size is only 586 Mb [20], which accounts for only approximately 22% of the total genome (about 2.57 Gb estimated by flow cytometry) [21]. That is, genome coverages were too low. Therefore, the clean reads were mapped using the ‘mem’ algorithm of BWA v0.7.12 [22] against the reference genome (all RAD-tags of the eleven *L. multiflorum* varieties) with the parameter -k 32 -M, and -M is an option used to mark shorter split alignment hits as secondary alignments [22]. The alignment results were marked using Picard (v1.129) (http://sourceforge.net/projects/picard/). Finally, variant calling of SNPs was performed for all samples using the Unified Genotyper of GATK [23]. The SNPs were filtered using GATK’s Variant Filtration with proper standards (-Window 4, -filter "QD < 2.0 || FS > 60.0 || MQ < 40.0 ", -G_filter "GQ < 20"). Variant allele frequency was used for downstream analysis; 1|1 is for homozygous mutation, 0|1 is for heterozygous mutation, 0|0 represents consistent with the reference sequence, NA represents other types and the file format was VCF.

### Data analysis

The SNPs after screening were used to calculate the effective number of alleles (Ne), the mean number of alleles (Na), the observed heterozygosity (Ho), the expected heterozygosity (He), and the Molecular Variance Analysis (AMOVA) by GeneAlex [24]; Nei’s genetic diversity (Nei) and Shannon’s information index (Shi) were determined by POPGENE (version 1.31, http://www.ualberta.ca/~fyeh/popgene). The larger parameter value of Ne, Na, Ho, He, Nei and Shi indicated that the varieties had the higher genetic variability among three bulked samples. In addition, POPGENE was used to calculate gene flow (Nm) between different varieties. The distinctive capacity was evaluated based on the fixation index (*F*_*st*_) between pairwise varieties [25]. Essentially, for *F*_*st*_ in the range of 0 to 1, there was no genetic differentiation between varieties when *F*_*st*_ was 0, and the highest genetic differentiation occurred when *F*_*st*_ was 1 [26]. In this study, the *F*_*st*_ value between 11 Italian ryegrass varieties was calculated by the Arlequin software [27]. In addition, the genetic diversity analysis within each variety was performed by calculating Nei’s nucleotide diversity (Pi) indices through the DnaSP [28] software, and genetic relationships within varieties were assessed by comparing the Pi values within bulks. The lower Pi value suggested lower diversity and better internal consistency [29].

Principal component analysis (PCA) is one of the most widely used statistical multivariate methods, which transforms the intricate correlated variables into simple correlated variables to perform the correlations among the varieties by multifactor dimensionality reduction [30]. Herein, based on the SNP variations of eleven varieties, PCA was performed based on the difference of SNPs of eleven varieties using the R package *adegenet* [31, 32]. Meanwhile, the discriminant analysis of principal components (DAPC) was performed using the *adegenet* package in R 3.5.3 [31]. The Genetic Structure provides an estimate of allele frequencies in each group and population relationships for every individual for a given number of clusters (K) [33]. In this study, a Bayesian clustering method was utilized to identify the genetic structure of the *Lolium multiflorum* varieties using the STRUCTURE program version 2.3 [34]. This analysis was performed under an admixture model that assumed independent allele frequencies and used 10,000 burn-in cycles followed by 100,000 Markov chain Monte Carlo iterations[34]. The batch run function performed a total of 110 runs (10 runs each for 1–11 clusters). The best value of the number of clusters (K) was determined for the *L. multiflorum* varieties using the the modal value of ∆K (K = 2–11) [35]. Furthermore, the maximum likelihood (ML) algorithm method was applied to construct the phylogenetic tree by MEGA 7.0 [36] (https://www.megasoftware.net/),, and the kinship relatedness matrix was calculated based on the VanRaden algorithm in GAPIT [37].

Finally, in order to estimate the fewest number of markers for variety distinction, the bootstrap re-sampling of 10, 20, 50, 80, 100, 500, 1000, 5000 and 10,000 markers were carried out. Meanwhile, the *P* values of AMOVA and Φ_ST_ were calculated for each re-sampling operation.

## Results

### Discovery of SNPs and genetic diversity analysis

All 33 bulks of the 11 tested Italian ryegrass varieties were sequenced using the Illumina HiSeq2000 platform. After quality filtering, 240.38 Gb of clean data were generated with the average of 7.28 Gb reads per bulk. Following rigorous screening (standards: MAF < 0.05 and integrity > 0.95), a total of 18,558,247 SNPs were detected from the 11 Italian ryegrass varieties, and several genetic diversity indexes were calculated. For all of these varieties, the Ne ranged from 1.2640 to 1.3237 with an average value of 1.2947. The Ho and He varied from 0.1546 to 0.1842 and 0.1505 to 0.1847 with an average of 0.1722 and 0.1861, respectively (Table 2). The Shi values ranged from 0.2207 to 0.2708 with an average of 0.2465 (Table 2). The Ho and He among varieties were also calculated to assess whether genetic diversity varied among varieties. The ‘TG’ variety had the highest Ne, He, Nei, and Shi values among all varieties, indicating that the genetic diversity of this variety was the highest with the maximum genetic variation within varieties, whereas these five parameters were the lowest for the ‘JT’ variety. This showed exactly the opposite results of the genetic diversity of ‘TG’ variety, as its genetic variation within varieties of ‘JT’ was lowest. Moreover, the Ne, Ho, He, Nei, and Shi values of the ‘CJT’ variety were lower than other varieties except ‘TG’. In other words, the internal consistency of ‘JT’ and ‘CJT’ varieties were better, while that of the ‘TG’ variety was the exact opposite.

**Table 2:** Genetic diversity indexes of eleven Italian ryegrass varieties

Variety	Number of bulks	Ne	Na	Ho	He	Nei	Shi
ABD	3	1.2808	0.6038	0.1688	0.1600	0.2040	0.2340
AGS	3	1.2771	0.6022	0.1679	0.1582	0.1971	0.2321
BD	3	1.3120	0.5528	0.1767	0.1782	0.2232	0.2613
CJT	3	1.2678	0.6199	0.1546	0.1524	0.1905	0.2231
DBR	3	1.3145	0.5510	0.1773	0.1794	0.2247	0.2629
GX	3	1.2959	0.5751	0.1775	0.1692	0.2114	0.2481
JT	3	1.2640	0.6227	0.1657	0.1505	0.1871	0.2207
LTT	3	1.2759	0.6065	0.1591	0.1572	0.1965	0.2304
SNF	3	1.3156	0.5465	0.1824	0.1804	0.2260	0.2647
TG	3	1.3237	0.5371	0.1796	0.1847	0.2315	0.2708
YC	3	1.3140	0.5493	0.1842	0.1794	0.2241	0.2632
Means	3	1.2947	0.5788	0.1722	0.1681	0.2106	0.2465
all	33	1.4402	0.0219	0.1538	0.2753	0.2916	0.4304

*Ne* Effective number of alleles, *Na* Mean number of alleles, *Ho* Observed heterozygosity, *He* Expected heterozygosity, *Nei* Nei’s genetic diversity, *Shi* Shannon’s information index

### Analysis of genetic differentiation index (*Fst*), nucleotide diversity (Pi) and AMOVA

The mean *Fst* values among the 11 tested Italian ryegrass varieties ranged from 0.1069 to 0.2480 (Table 3), which revealed that significant genetic differentiation existed among them*.* The lowest *Fst* values (0.1069) were detected between the ‘YC’ and ‘TG’ varieties, which showed that these had the lowest genetic differentiation and differences. Conversely, the highest *Fst* values (0.2480) were presented between the ‘CJT’ and ‘AGS’ varieties, which had the highest genetic differentiation and differences.

**Table 3:** Genetic differentiation coefficients (*Fst*) among different varieties

Variety	AGS	BD	CJT	DBR	GX	JT	LTT	SNF	TG	YC
ABD	0.1232	0.2038	0.2377	0.1908	0.2122	0.1418	0.1647	0.2037	0.1828	0.1933
AGS		0.2098	0.2480	0.1889	0.2169	0.1407	0.1713	0.2007	0.1873	0.2031
BD			0.1917	0.1336	0.1504	0.2267	0.1966	0.1491	0.1448	0.1450
CJT				0.1788	0.1431	0.2519	0.2188	0.1565	0.1726	0.1184
DBR					0.1593	0.1956	0.1636	0.1220	0.1220	0.1345
GX						0.2349	0.1994	0.1353	0.1540	0.1129
JT							0.1824	0.2087	0.1818	0.1974
LTT								0.1625	0.1751	0.1789
SNF									0.1371	0.1211
TG										0.1069

All of the *Fst* values were significantly different at *P* < 0.05

**Fig. 1:**
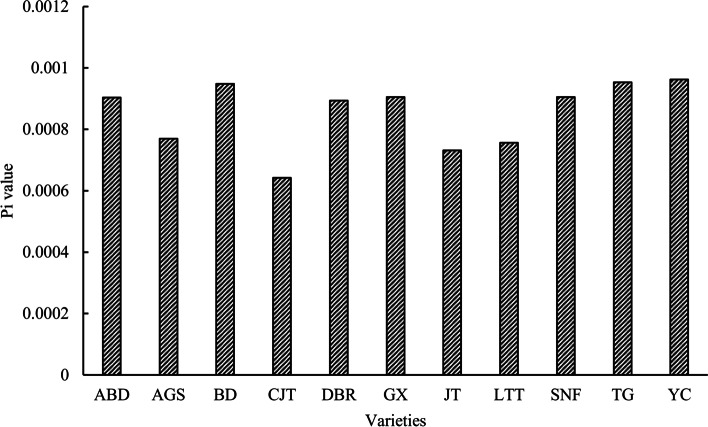
Analysis of genetic diversity of varieties based on the number difference of SNPs

The nucleotide diversity (Pi) analysis within the tested Italian ryegrass varieties was able to reflect the variety consistency at the molecular level. The results showed that the ‘CJT’ had the lowest Pi value (Fig. 1), indicating that it had the best concordance among the tested varieties. This result was similar that from the other genetic indexes (Table 2).

The AMOVA analysis was used to evaluate the genetic variance within varieties, which revealed 37.27% of the genetic variance among varieties, and 62.73% of the genetic variance within varieties (Table 4). The AMOVA (Φ_ST_ = 0.373) also supported the varieties’ divergence based on Shannon’s information index (0.430, Table 2). Thus, a large variation was indicated among varieties. Nevertheless, the high variation among bulks remained within varieties may pose difficulties for identifying these 11 genetically heterogenous varieties.

**Table 4:** Hierarchical partitioning of genetic variance using AMOVA

Source of Variation	Degrees of Freedom (df)	Sum of Squares (SS)	Variance Components (VC)	MS	Percentage of Variation (%)
Among varieties	10	0.600671997	0.0056253	0.060067	37.27%
Within varieties	22	0.514943428	0.0094685	0.023407	62.73%
Total	32	1.115615425			*p* < 0.001*

### DAPC, PCA and genetic structure analysis

**Fig. 2:**
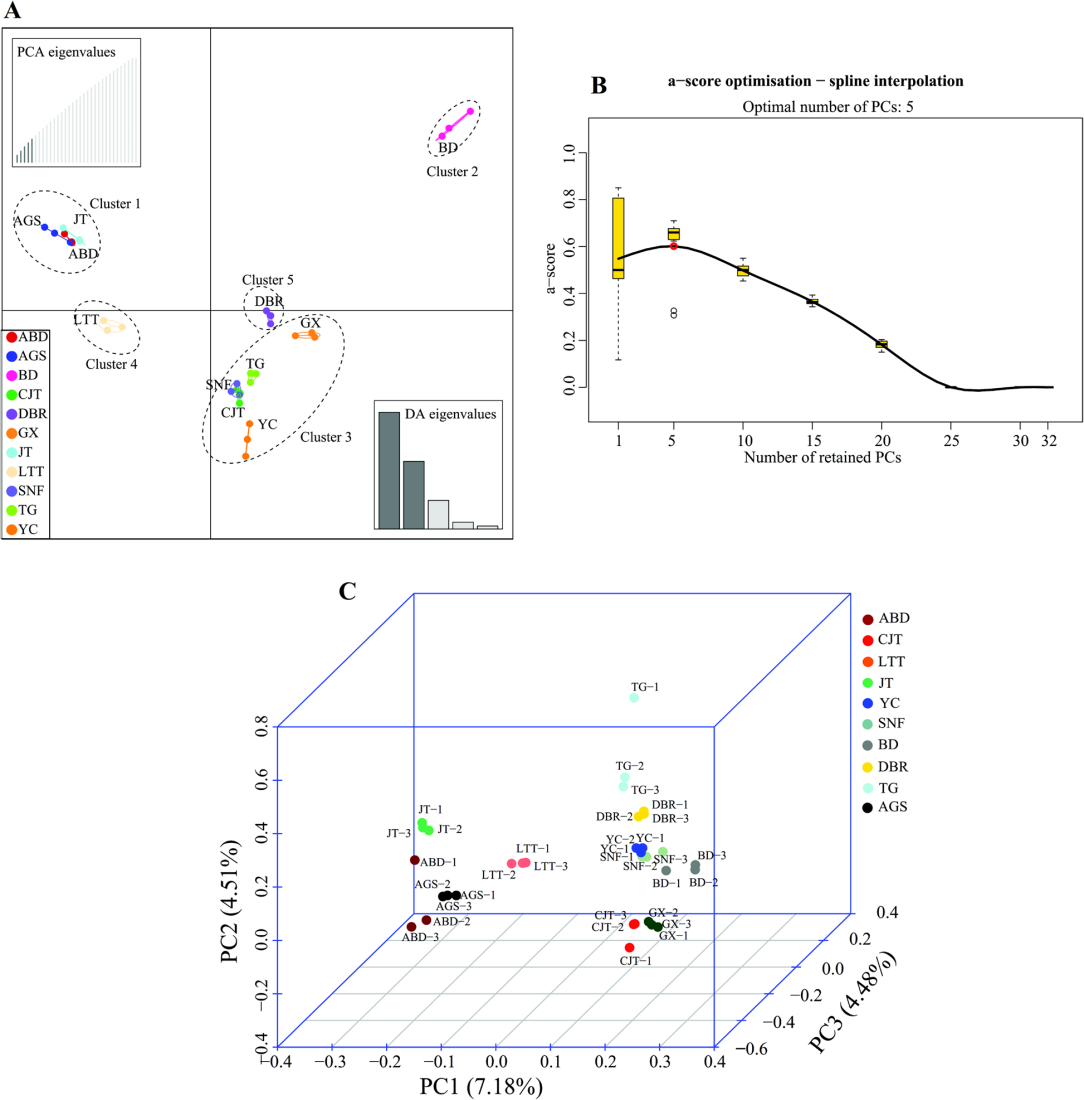
**A** Discriminant analysis of principal component (DAPC) results of the SNP data for Italian ryegrass varieties. The axes represent the first two linear discriminants (LD). Each dot represents a bulk. **B** The spline interpolation of score optimization of DAPC and the optimal number of PCs were 5. **C** Principal component analysis (PCA) based on the number difference of SNPs. The different colors of the dots represent the different varieties, and the three same color of dots represent the different bulks of the same varieties

The DAPC analysis based on SNP data grouped the 11 tested Italian ryegrass varieties into 5 clusters (Fig. 2B). Cluster 2 and 4 were clearly differentiated, which showed that ‘LTT’ and ‘BD’ belonged to disparate subgroups. Bulks in the other three clusters had a distinct division (Fig. 2A). The ‘AGS’, ‘ABD’ and ‘JT’ varieties had similar genetic structures. The principal component analysis (PCA) indicated that the distributions of most varieties were totally separated from each other except those of ‘ABD’ and ‘AGS’ (Fig. 2C), suggesting a strong gene flow and a close genetic relationship between them (Table S1). These genetically similar varieties could be further differentiated by other methods. The results were consistent with those of genetic structure and *F*_*ST*_ analysis.

**Fig. 3:**
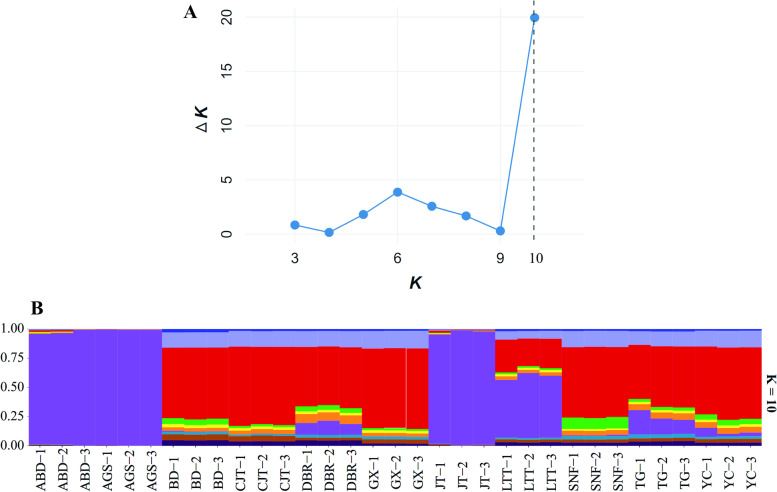
Genetic structure of *L. multiflorum* varieties for a K = 10 population model; **A** The values of ΔK plotted over ten runs for each K value, where the best K value was 10. **B** Structure stack diagram of 33 bulks of varieties for K = 10

In the Bayesian assignment analysis performed by STRUCTURE, the ad hoc ∆K statistics exhibited a signal of at best K = 10 indicating that 11 Italian ryegrass varieties consist of ten genetic clusters (Fig. 3A and B). The greater proportion of an assignment that a variety bulk received, the greater the possibility that the assignment belonged to the corresponding genetic background. To elucidate the main genetic structure and composition, bulk sample of varieties that were assigned to a single cluster with more than 70% similarity were defined as pure groups. Of 11 varieties, only 3 could be assigned to pure clusters based on the 70% membership threshold. Meanwhile, three bulks from the same variety tended to cluster clearly together. Three PVP varieties ‘ABD’ ‘AGS’ ‘JT’ has a pure genetic background due to membership more than 0.7. Except for these three varieties, the different matrix structural constituent of background indicated that 24 bulks of the other 8 Italian ryegrass varieties could be clearly distinguished by the STRUCTURE analysis.

### Phylogenetic and kinship analysis

**Fig. 4:**
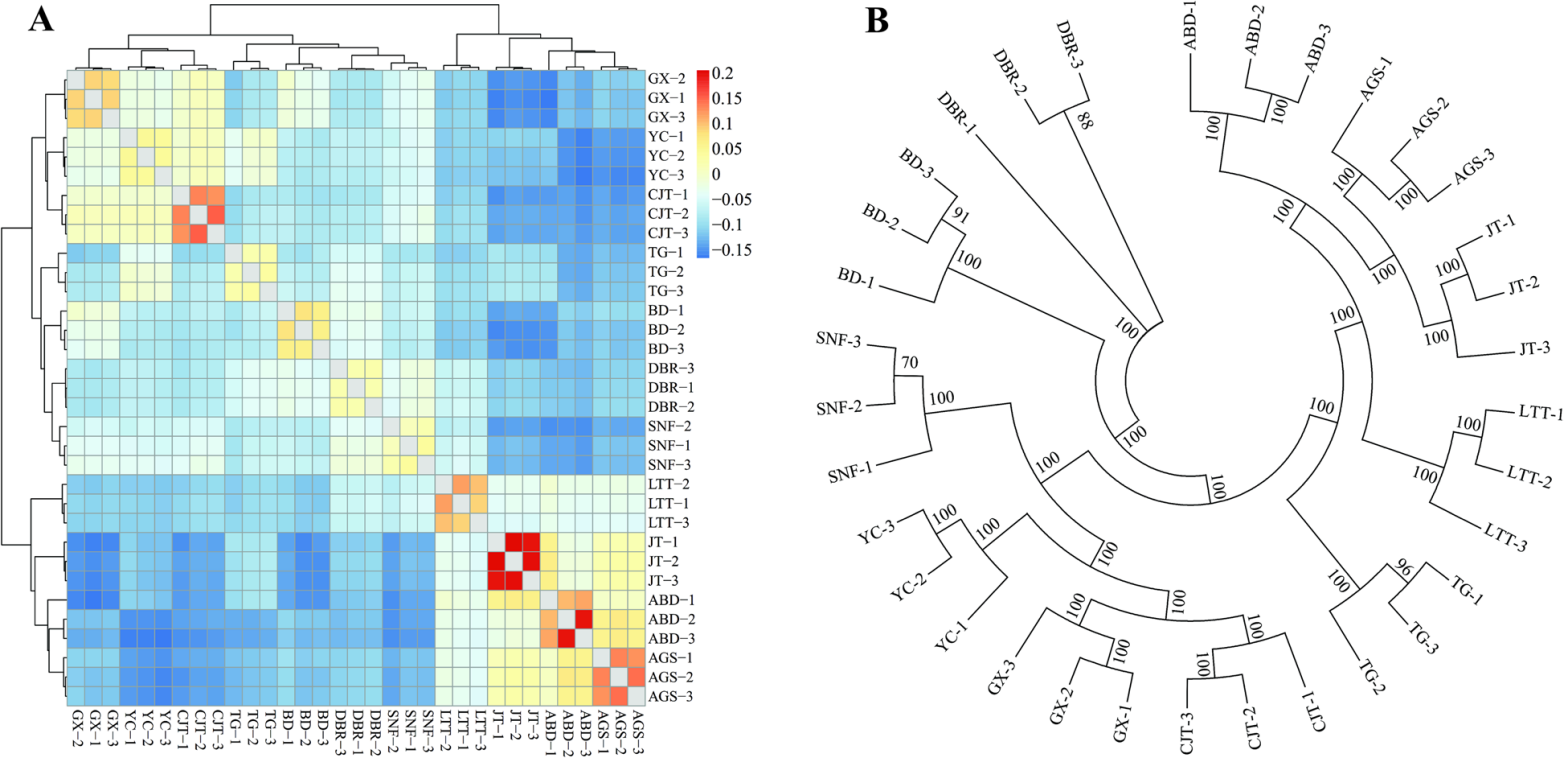
Kinship matrix for the eleven Italian ryegrass varieties (**A**). Establishment of phylogenetic tree by the maximum likelihood method **(B**). Numbers at the nodes represent confidence levels of bootstrap analysis with 1000 replications as a percentage value

The stepwise kinship matrix was calculated based on the obtained SNPs, and all the varieties were clearly distinguished, that is, all 33 bulks were grouped into 11 subgroups (Fig. 4A). Three bulks of each variety were highly related, which indicated that the three bulks were quite reproducible. In short, the 11 tested varieties were clearly distinct. The maximum likelihood (ML) algorithm method was used to construct the phylogenetic tree among the 11 Italian ryegrass varieties (Fig. 4B). The findings showed that three bulks of each Italian ryegrass varieties were clustered together, and thirty-three bulks could be distinguished completely by the number of varieties. Compared with DAPC, PCA, and genetic structure analysis, the phylogenetic tree proved a better alternative to distinguish varieties based on RAD-seq.

### Distinguish ability of SNP markers in varieties

**Fig. 5:**
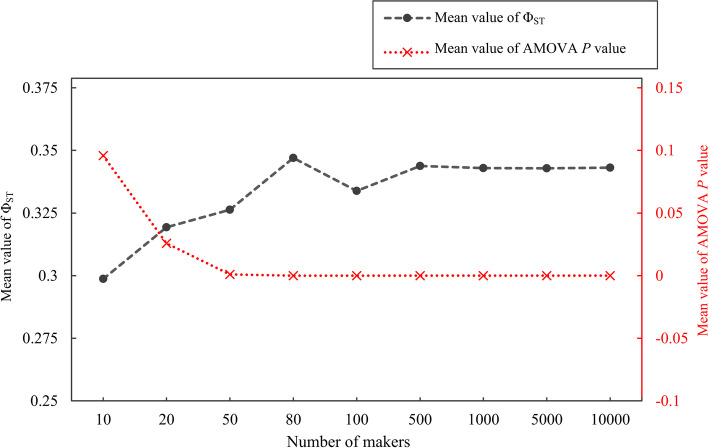
Effect of number of markers from 10 to 10,000 on the mean Φ_ST_ value (left axis) and the mean AMOVA *P* value (right axis) between the ‘TG’ and ‘YC’ varieties

The calculated values of AMOVA between the two least significantly different varieties were used to test the ability to distinguish different varieties. All *F*_ST_ values of the eleven varieties were significant (*P* < 0.05), and the *F*_ST_ values between pairs of varieties (Table 3) ranged from 0.1069 (between ‘TG’ and ‘YC’) to 0.2480 (between ‘CJT’ and ‘AGS’). The most similar varieties ‘TG’ and ‘YC’ were selected, for which 100 samplings of the 10, 20, 50, 80, 100, 500, 1000, 5000 and 10,000 markers were performed for the AMOVA. The average *P* values of the AMOVA-based Φ_ST_ decreased along with the increase of the number of markers (Fig. 5), and the *P* value reached the minimum at 50 SNPs. The mean Φ_ST_ value rose as the marker number increased when the number of markers was 10 to 80. Meanwhile, the Φ_ST_ value reached the maximum at 500 SNPs. As a result, according to Fig. 5, we suggested that it was adequate to distinguish the varieties using 500 SNPs.

## Discussion

### RAD-seq possesses powerful identification ability in *L. multiflorum* varieties

The accurate distinction of varieties plays a crucial role in international intellectual property protection. Over the past few decades, the DUS tests and traditional molecular markers have been widely used for variety identification, and they proved effective for the distinction of self-pollinating plants [6, 38]. For plants with outcrossing mating systems, however, within-variety variation is usually high because pollen can be widely spread between varieties, which leads to a low level of differentiation between them [39]. Therefore, it has been difficult to distinguish these varieties, especially the species with a vast number of varieties, relying solely on DUS tests by morphological traits or traditional low-throughput molecular markers. In contrast, high-resolution and high-throughput approach sequencing methods are more convenient and reliable for this task. In our previous study, SSR markers were used to distinguish 6 Italian ryegrass varieties [12]. We found that 17 out of 29 polymorphic SSR markers could not completely distinguish the 6 tested Italian ryegrass varieties. In the present study, 11 Italian ryegrass varieties were successfully distinguished by high-throughput SNP genotyping from RAD-seq. Therefore, SSR markers could be more effective than SNP markers in routine genetic diversity analysis [40], whereas this is not necessarily the case in the varietal identification. Satisfactory repeatability of three bulks from one same variety were observed from kinship analysis and STRUCTURE analysis showing extremely high similarity among the three bulks. 11 Italian ryegrass varieties were clearly differentiated by SNP markers by phylogenetic and kinship analysis based on RAD-seq, whereas they were distinguished by 30 DUS traits from the vegetative to the reproductive stage for these varieties. Therefore, we think that the RAD-seq method is more discriminative than SSR markers. For instance, SNP markers is considered as more efficient than traditional SSR markers in alfalfa [4, 41] and soybean [6] varieties identification.

Traditional molecular markers are basically limited to dozens of pairs of primers, of which only a few hundred bands can be amplified based on PCR amplification [1, 8, 12]. In contrast, 18,558,247 SNP markers were detected in this study, which proves that RAD-seq could detect far more sites than traditional molecular markers. Thus, RAD-seq has much higher genome coverage than traditional molecular markers. In this study, the identification capacity of Italian ryegrass varieties reached a peak when 500 SNPs were used, which is fewer than that for the lucerne (*Medicago sativa*) based on GBS (where at least 1,000 SNPs were needed) [4]. It may be argued that RAD-seq is superior to GBS for cultivar identification characterization. It should be also noted that varietal identification efficiency might be affected by different sequencing platforms and bioinformatics approaches, such as sequencing depth and genomic reference sequence.

In addition to optimal high-throughput sequencing, a suitable sampling strategy was also essential for the success of variety identification of OPVs. Previous studies [1, 12, 42] used mixed samples for variety distinction to keep track of the genetic diversity, and these could greatly facilitate evaluating their performance [43], however, there were only 10–30 individuals for a bulked sample, which did not sufficiently represent all variation in the population. In addition, for a traditional molecular marker, the amount of DNA template used is insufficient if there are too many individuals in a mixed sample, and the lower DNA content of each individual might lead to weak PCR amplification patterns inadequately representing the whole sample. Moreover, primers of traditional molecular markers might compete in DNA binding sites when a large number of DNA templates exist in a mixed sample [1, 12]. On the contrary, high-throughput RAD-seq does not present this problem. Therefore, we used 150 individuals to compose three bulks per a single annual ryegrass variety, which showed a good distinction performance.

### Prospects of genotyping in variety registration

Despite the present limitations, the current Plant breeders' rights (PBR) protect or plant variety protection (PVP) of registering candidate varieties still relies on DUS testing, which applies morpho-physiological traits examination to indicate whether candidates are distinct (uniform and stable) from all existing registered ones [44]. However, with the rapid growth in numbers of registered herbage varieties, effective variety characterization becomes increasingly challenging in major outbreeding forage species such as annual ryegrass. Thus, some new candidates could not sufficiently differentiate from that existing leading varieties, and consequently lead to DUS rejections. The genetic diversity analysis based on high-throughput molecular markers has become new approach for herbage DUS testing [45] Similar to the present study about annual ryegrass variety identification, the successful use of GBS-SNP markers on bulked plants to discriminate herbage varieties due to cost-saving strategies has been reported for perennial ryegrass [16] and alfalfa [4, 46]. These findings also revealed higher discrimination power by genotype examination than standard DUS testing when using appropriate analytical methods of diversity. Meanwhile, it has been found that that marker resolution of varietal discrimination increased as the number of SNP loci increased [16]. Therefore, after DUS testing by morphophysiological characters, it is very necessary to perform high-throughput marker analysis on the candidate registered varieties when their phenotypes are not significantly different from existing leading varieties [4]. Moreover, if molecular marker of candidate genes closely linked with DUS morpho-physiological traits were identified, they may have a great potential as an effective alternative to DUS characters. Another particular concern of variety discrimination by molecular markers is that there is no evidence-based, clear-cut threshold to judge whether candidate varieties be distinguished from existing popularized varieties, especially for those EDVs (Essentially Derived Varieties) with extremely similar molecular identities [47]. Consequently, UPOV (www.upov.int) argues against the widespread use of molecular marker-based DUS testing to protect Plant breeders' rights (PBR) for any species. Recently, a proposal named “vmDUS” (value-molecular DUS) test was implemented to require that the candidate cultivar has a clearly improved VCU (Value for cultivation and use) trait when compared to a genetically similar registered cultivar [7].

## Conclusion

In this study, the RAD-seq technique performed on bulked samples of 11 *L. multiflorum* varieties proved to be efficient in their distinction, thus is considered a valuable assistant measure for DUS testing. For certain varieties with similar genetic relationship that are therefore difficult to identify, the combination of RAD-seq and phenotypic evaluation is the recommended approach. Our findings can assist variety registration with proposing regulatory changes to incorporate the higher established variety distinction potential for *L. multiflorum*.

## Supplementary Information

**Additional file 1:** **Table S1.** Gene flow among different varieties.

## Data Availability

All data in this article are available. The data of RDA-seq are available at the CNGB (https://www.cngb.org/) and the accession number is CNP0001984.
