# Supplementary material for: RAD-seq as an effective strategy for heterogenous variety identification in plants—a case study in Italian Ryegrass (Lolium multiflorum)
Source: BMC Plant Biol. 2022 May 5;22:231. doi: 10.1186/s12870-022-03617-6 (PMC9069751; doi:10.1186/s12870-022-03617-6)
Supplement: Supplementary file 1 — Additional file 1: Table S1. Gene flow among different varieties. [file 12870_2022_3617_MOESM1_ESM.docx]

Table S1 Gene flow among different varieties

| Varieties | AGS | BD | CJT | DBR | GX | JT | LTT | SNF | TG | YC |
| --- | --- | --- | --- | --- | --- | --- | --- | --- | --- | --- |
| ABD | 3.7251 | 0.8347 | 1.2464 | 2.2690 | 0.8509 | 1.7231 | 3.6140 | 2.2403 | 1.1076 | 1.1014 |
| AGS |  | 0.6540 | 1.0284 | 2.0527 | 0.6643 | 2.9996 | 2.6302 | 2.1004 | 1.8614 | 1.7344 |
| BD |  |  | 1.3643 | 3.9554 | 0.8223 | 1.4730 | 1.7122 | 3.0229 | 2.5620 | 2.6453 |
| CJT |  |  |  | 1.0096 | 2.6258 | 0.7464 | 0.8052 | 1.2372 | 1.0148 | 1.6253 |
| DBR |  |  |  |  | 2.6803 | 1.2316 | 0.7314 | 0.9242 | 1.8658 | 1.6388 |
| GX |  |  |  |  |  | 1.5650 | 1.8826 | 2.1945 | 2.6390 | 2.2230 |
| JT |  |  |  |  |  |  | 1.3298 | 1.2121 | 0.6883 | 0.6529 |
| LTT |  |  |  |  |  |  |  | 0.7676 | 1.1681 | 1.2019 |
| SNF |  |  |  |  |  |  |  |  | 1.6397 | 1.9983 |
| TG |  |  |  |  |  |  |  |  |  | 0.9249 |
